# Real world outcomes of Fabry disease in Italian excellence centers: the ground study

**DOI:** 10.1186/s13023-026-04370-x

**Published:** 2026-05-30

**Authors:** Iacopo Olivotto, Renzo Mignani, Federico Umberto Pieruzzi, Antonino Tuttolomondo, Davide Noto, Maurizio Pieroni, Elena Verrecchia, Eleonora Ingrassia, Annarita Armaroli, Antonio Pisani

**Affiliations:** 1 Meyer Children’s Hospital IRCCS, Firenze, Italy; 2 Cardiomyopathy Unit, Careggi University Hospital, Firenze, Italy; 3 Department of Medical and Surgical Sciences (DIMEC), Alma Mater Studiorum - University of Bologna, Bologna, Italy; 4 Nephrology, Dialysis and Transplant Unit, IRCCS Azienda Ospedaliero-Universitaria di Bologna, Bologna, Italy; 5 Nephrology and Dialysis Unit, Infermi Hospital, Rimini, Italy; 6 Fondazione IRCCS San Gerardo dei Tintori, Nephrology, Monza, Italy; 7 School of Medicine and Surgery, University of Milano-Bicocca, Milan, Italy; 8 Department of Health Promotion, Mother and Child Care, Internal Medicine and Medical Specialties, Molecular and Clinical Medicine, University of Palermo, Palermo, Italy; 9 Department of Health Promotion, Maternal and Child Health, Internal and Specialized Medicine of Excellence “G. D. Alessandro” (PROMISE), University of Palermo, Palermo, Italy; 10 Department of Aging, Orthopedic and Rheumatological Sciences, Fondazione Policlinico Universitario”A. Gemelli” IRCCS, Roma, Italy; 11 Medical Affairs Chiesi Italy S.p.A, Parma, Italy; 12 Department of Public Health, Federico II University, Naples, Italy

**Keywords:** Fabry disease, MSSI, FASTEX, Enzyme Replacement Therapy

## Abstract

**Background:**

A better understanding of Fabry Disease (FD) in terms of diagnostic journey, patient profile and use of available treatments remains topical. The Global buRden and treatment trajectOries in Italian patients with Fabry disease: a retrospective longitUdinal and cross-sectioNal study-GROUND was designed to provide a comprehensive overview of a real-world FD Italian cohort.

**Methods:**

This was a retrospective, multicenter, longitudinal and cross-sectional study of adult FD patients of either sex with ≥ 3 years of follow-up. Severe/fatal events from birth to last visit was the composite primary outcome. Secondary outcomes were disease progression measured by FAbry STabilization indEX (FASTEX); disease activity assessed by clinically significant events/FD related symptoms; time from first manifestation to diagnosis; treatment trajectories.

**Results:**

199 patients, 73 males and 126 females, 169 classic and 30 late-onset phenotypes, were enrolled at 8 Italian centers. Mean time from first FD symptom to diagnosis was 5.03 years (range 0.00-43.90), with a median of 0.64 years (range 0-19.29) from diagnosis to start of FD specific treatment. Initial manifestations were chronic pain (18.1%), angiokeratomas (13.1%), acroparesthesia and abdominal pain (12.5%), or left ventricular hypertrophy (LVH) (8.5%). 143 missense/nonsense GLA variants were identified. 82% patients were actively treated with one of the FD specific therapies, 24% had switched ≥ 1 treatment, while 5% had definitively stopped treatment. The primary outcome was experienced by 31.7% of patients for a total of 85 severe/fatal events, mainly in the treated cohort: 20.6% had a cardiac event, 11.1% a cerebral event 7.5% patients experienced a renal event, while fatal events occurred in 7 patients (3.5%). 88.9% reported ≥ 1 clinically significant event, with detection/worsening of LVH being the most common (41.2%). Estimated Glomerular Filtration rate (eGFR) slopes in last 1–3 years of follow-up were − 2.07 ml/min/m^2^ (SE: 1.38) and − 0.76 and − 2.07 ml/min/m^2^ (SE: 1.75) in classic males and females, respectively. Long-term FASTEX identified 31.1% of patients as being unstable.

**Conclusions:**

Even though FD patients were promptly treated, severe clinical events occurred in about 30%, mainly classic males, highlighting the need for sensitive tools assessing outcomes and novel therapeutic options.

## Introduction

First described in 1898 by doctors J. Fabry and W. Anderson as a dermatological condition characterized by angiokeratoma in male subjects, Fabry disease (FD) is an X-linked inherited lysosomal disorder that results from the deficient activity of the lysosomal glycohydrolase α-galactosidase A (α-Gal A) which is coded by the galactosidase alpha (*GLA)* gene on Xq22 [1–3]. Over 1,000 variants in the *GLA* gene (i.e., missense, or nonsense, or splicing mutations, small or large deletions, insertions) have been recognized as altering synthesis, trafficking, folding, degradation and overall activity of α-Gal A, thus affecting disease severity [4]. The enzymatic defect leads to a progressive accumulation of globotriaosylceramide (Gb3) and related glycosphingolipids, particularly in the endothelium of blood vessels in the skin, kidney, heart, brain and in smooth muscle cells resulting in cellular dysfunction, followed by a structural damage that, over a variable period of time, leads to progressive vascular occlusion, ischemia and ultimately organ impairment or failure in the most severe cases [5]. FD patients (pts) suffer from a multisystemic disease with progressive dialysis-dependent renal insufficiency, hypertrophic cardiomyopathy, recurrent strokes, gastrointestinal (GI) pain, and neuropathic pain of the extremities [6]. Current newborn screening disease prevalence ranges from 1:1,368 to 1:8,882 in males and from 1:6,000 to 1:40,000 among female carriers, and given that FD is an X-linked disorder, family history and clinical traits in the proband and relatives are highly informative [7–10]. All hemizygous male subjects are affected, even though signs and symptoms can be present also in female individuals, due to the random inactivation of one of the two X chromosomes in each cell during embryogenesis (i.e., lyonization), which leads to a greater clinical variability in women than in men [11]. The condition is categorized into classic (Cl) and non-classic (NCl)/late-onset phenotypes, with a wide variability as to age of onset and clinical features [12]. In the Cl phenotype, no α-Gal A activity is generally detected, even though with sensitive enzyme assay methods a 1–3% residual activity has been reported [13]. Hemizygous Cl males typically develop signs and symptoms in childhood or adolescence (i.e., delayed puberty and growth, GI symptoms, cornea verticillata, angiokeratomas, acroparesthesias/neuropathic pain), severe organ complications as early as their 20–30 s, leading to renal, cardiac, cerebrovascular disease, until premature death [14]. Either sex NCl pts show a variable residual α-Gal A activity between 2 and 20%, and they generally suffer from a later and milder form of the disease [15]. In males, the determination of α-Gal A activity in blood leukocytes or from dried blood spots (DBS) is the method of choice for diagnosis confirmation, while molecular genetic testing demonstrating a disease-causing variant in the *GLA* gene is necessary for diagnosis in female subjects [16]. If not treated, FD is associated with significant mortality and morbidity, with a 20- and 10-year reduction of life expectancy in males and females, respectively, *versus* the general population, mostly due to end-stage renal disease, and cardiovascular complications [17]. Available FD treatments may prevent irreversible organ damage, thus a timely and accurate diagnosis awareness is of utmost importance, and symptomatic treatment has gradually shifted to a more integrated approach using FD-specific agents, including enzyme replacement therapies (ERTs) [18]. Although the overall outcome of the disease has improved, several challenges remain to be addressed, such as a low disease awareness among physicians, while clinical and treatment approaches show disparities among countries. Once the diagnosis is established, the phenotypic and genotypic characterization must be addressed, and disease monitoring by means of reliable biomarkers is needed to delineate disease severity and progression [19]. In addition, disease burden is hard-to-estimate, and side effects of available treatments along with an unpredictable disease progression may affect patients’ Quality of Life (QoL) [20]. Since ERTs are limited by clinical variation and variable incidence of infusion-related reactions, oral chaperone treatment represents an alternative, but its use in case of amenable mutations only, along with some limitations in its therapeutic efficacy, limit its broader applicability [21].

The Global buRden and treatment trajectOries in Italian patients with Fabry disease: a retrospective longitUdinal and cross-sectioNal study-GROUND study (NCT04916977) addresses the current hot topics in FD by describing a large Italian population of patients in a real-life setting, delineating the burden of the condition, the delays in its diagnosis, and predictors for disease progression.

## Materials and methods

### Study design and objectives

**Fig. 1:**
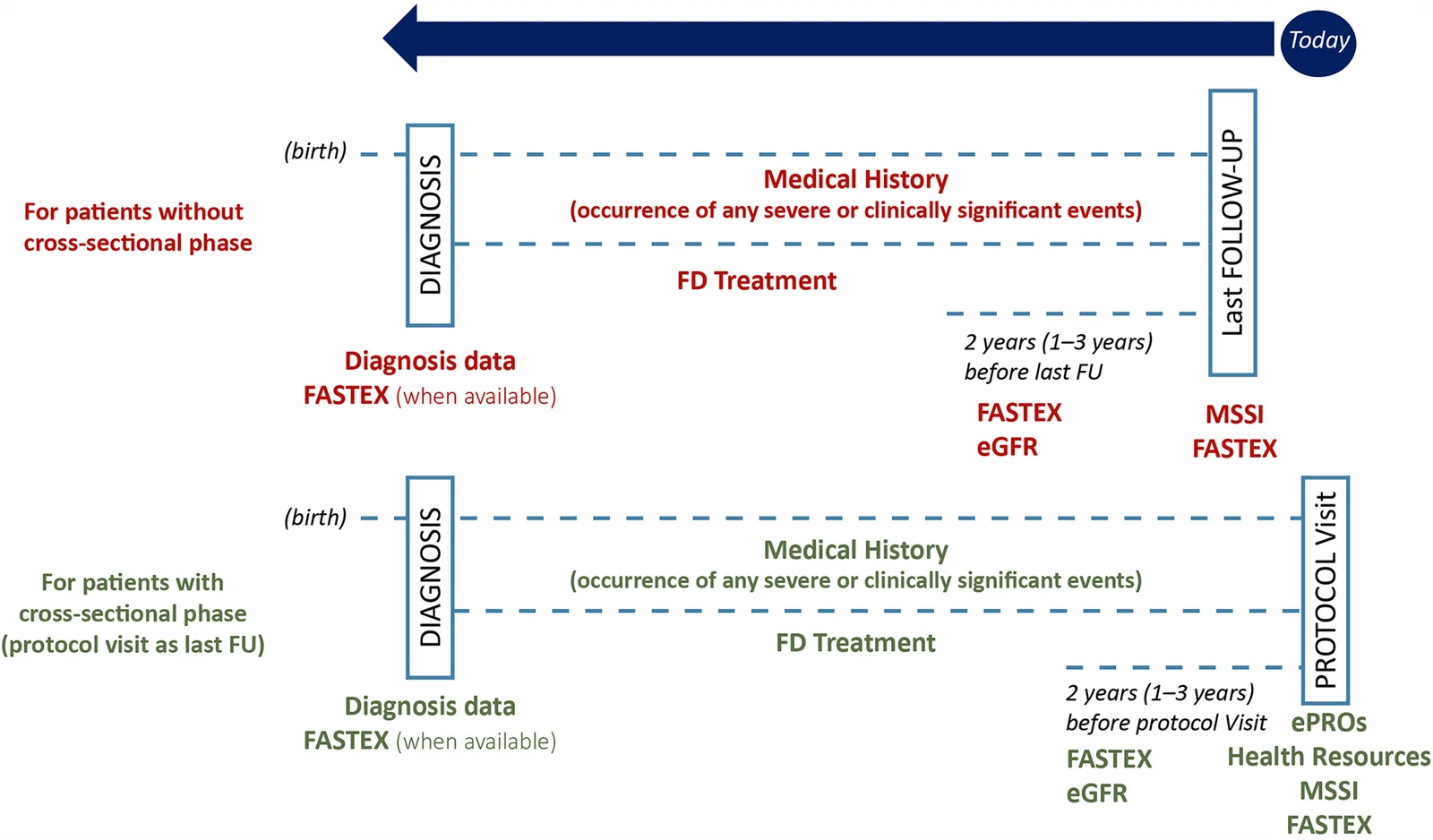
Study design of the GROUND study. FD, Fabry disease; Electronic patient-reported outcomes, ePROs; Estimated glomerular filtration rate; eGFR; FD, Fabry Disease; FAbry STabilization indEX, FASTEX; Mainz Severity Score Index, MSSI

This was a multicenter, retrospective, longitudinal cohort study with a final cross-sectional evaluation. In the retrospective phase, clinical data before and after the diagnosis were collected until the last follow-up (LFU) upon consent, and a cross-sectional visit (CSV) was planned to evaluate actual clinical data. Adult pts of either sex, living or deceased, ≥ 18 years of age with a documented FD diagnosis, and ≥ 3 years of FU or early occurrence of severe/fatal outcomes were included in the study (Fig. 1).

Co-primary endpoints were:

- Time to first occurrence of severe and fatal outcomes derived by event free survival from birth to LFU or CSV. In particular, first occurrence of severe and fatal outcomes, defined as the first event that occurred among renal, cardiac, cerebral, or fatal event (i.e., whichever occurred first) were considered, and they were also analysed individually as time to first renal event, time to first cardiac event, time to first cerebral event, time to fatal event.
- The comprehensive burden reflecting disease severity, considered as a measure of extension of clinical impairment, as assessed by median Mainz Severity Score Index (MSSI) at LFU or CSV [22].

Secondary endpoints were as follows:

- Percentage of pts with disease progression measured by FAbry STabilization indEX (FASTEX) and defined as a change > 20% from the first available FASTEX score to the LFU/CSV and in the last 2 years until LFU [23];
- Percentage of pts with disease activity assessed by the occurrence rate of clinically significant events from diagnosis to LFU/CSV by analyzing separately the following events: decline in renal function, detection/worsening of LHV, increase in lysoGb3 and occurrence of new Fabry-related symptom, recurrence of Fabry-related symptoms at any time.
- Evolution of burden evaluated by the slope of renal deterioration in the last three years, as well as number, length, and reason of hospitalization, number and type of clinical/instrumental visit, as well as non-hospital service accesses in the last year;
- Time from first manifestation to diagnosis and from diagnosis to first comprehensive evaluation with first FASTEX score available, along with the number and type of healthcare professionals (HCPs) involved in diagnosis;
- Predictors of occurrence of events, MSSI and FASTEX change;
- Percentage of pts prescribed a different treatment among ERT *naïve* and switcher groups, with determinants of untreated, treated discontinued, and treated persisting *status*.

### Statistical methods

All patients meeting inclusion criteria were included in the retrospective phase, written informed consent was required for participation in the cross-sectional visit and for retrospective genetic/clinical data collection, while for deceased or untraceable patients only non-genetic retrospective data were collected without consent, in compliance with Italian regulations, and they did not undergo cross-sectional evaluation or contribute additional outcomes. As to primary outcomes, time to occurrence of first severe renal, cardiac, cerebral and fatal clinical outcomes separately, as well as any first event from birth to LFU or CSV (i.e., composite outcome) was analyzed by means of the Kaplan-Meier (KM) method, log-rank tests were applied to test the difference between groups by phenotype and treatment *status*, and Cox Proportional Hazards Regression (CPHR) model was used to assess the potential influence of some patients’ characteristics on the occurrence of severe and fatal outcomes. Descriptive analysis was performed for MSSI score components, i.e., general, neurological and psychiatric/psychosocial, cardiovascular and renal. As to secondary outcomes, FASTEX score was assessed during the CSV/LFU and retrospectively calculated at the diagnosis or as soon as available, and 2 years before LFU, or when available within 1–3 years before LFU. The number and percentage of pts with disease progression were descriptively analyzed considering FASTEX score as a continuous variable. Analysis of variance (ANOVA) for repeated measures was performed, using all the scores available in the last 1–3 years until the LFU/protocol visit as dependent variable, while time points were included in the longitudinal model as repeated factor. Potential predictors of clinically significant changes in FASTEX score were analyzed using multivariate logistic regression, and correlation among FASTEX change in the last 1–3 years until the LFU/protocol visit and variables collected at the LFU or CSV was assessed using Spearman coefficient, polyserial, or polychoric correlation. The number and percentage of pts with disease activity was assessed by the occurrence rate of clinically significant events from diagnosis to LFU/CSV. Decline in renal function, detection/worsening of LVH, myocardial replacement fibrosis, increase in lysoGb3 or other biomarkers, and reoccurrence or occurrence of new FD-related symptoms at any time were analysed together and separately, and type of symptom was examined. Descriptive analysis of units nmol/L and ng/ml of LysoGb3 was performed when its increase was reported as clinically significant. Decline in renal function was descriptively analysed using frequencies and percentages, and it was used to assess evolution of burden from LFU/CSV; moreover, an ANOVA for repeated measures was performed, using estimated glomerular filtration rate (eGFR) values in the last 1–3 years until the LFU as dependent variable. Time from first FD symptom to diagnosis was analysed by KM method, and potential predictors of time were explored using the CPHR model. Health-related resource consumption was analysed by means of the Poisson regression model as to number, duration and reason of hospitalizations, number of emergency room (ER) visits, outpatients’ visit, instrumental or laboratory examinations, and non-hospital service accesses in the last year from LFU or CSV. Age, sex, smoking habits, family history of FD, disease duration, comorbidities, symptoms at diagnosis, genetic features, phenotype categorization and treatment *status* were investigated as potential predictors of secondary endpoints.

## Results

Overall, 200 pts were screened, since one was excluded for personal reasons 199 were enrolled, and 49% of pts came from one of the 8 centers involved. Informed consent was signed by 88% of pts (*n* = 175) who underwent the cross-sectional visit, among those who did not sign 47% (*n* = 17) were not-traceable, 20% (*n* = 7) were deceased, while in 33% (*n* = 12) of the cases the reason was not specified.

### Baseline population characteristics

Main population features are summed up in Table 1 and described as follows:

- Study population included 126 females (63.3%) and 73 males (36.7%). The mean age was 46.7 (SD 16.3) years (median 49.0 years, range 18–82 years). All subjects were of white ethnicity, except 1 African American and 1 patient reported as “*other ethnicity*”. The overall population included 35 (17.6%) current smokers, 18 (9.0%) ex-smokers, and 146 (73.4%) non-smokers.
- As to phenotype, 15% of pts (*n* = 30) were classified as NCl pts, while 85% (*n* = 169) as Cl pts. The proportion of female subjects was higher among NCl pts than Cl pts (70.0% vs. 62.1%, respectively), with reversed rates for males (30.0% and 37.9%, respectively). The differences between Cl pts and NCl pts were not statistically significant as to age, gender, ethnicity, and smoking habits.
- Treated (Tr) pts showed a slightly higher mean age than non-treated (NTr) pts (47.4 years vs. 43.4 years, respectively); the proportion of female subjects was higher in NTr pts than in Tr pts (88.9% vs. 57.7%, respectively), with reversed rates for males (11.1% and 42.3%, respectively), and the difference in gender distribution between the two subgroups was statistically significant (*p* < 0.001).
- Overall, 90.5% of pts had ≥ 1 relative suffering from FD, in a similar proportion among Cl pts and NCl pts (89.9% vs. 93.3%, respectively), as well as among Tr pts and NTr pts (89.6% vs. 94.4%, respectively); the overall mean number of relatives suffering from FD was 2.5 (SD 1.5).
- Overall, 61.3% of pts had ≥ 1 concomitant disease. Most commonly reported comorbidities were vascular disorders (33.7%), hypertension (32.7%), dyslipidemia (20.1%), and endocrine disorders (16.1%) in particular hypothyroidism (8.0%).
- Overall, 54.8% of pts were using ≥ 1 concomitant medication, mainly agents for the cardiovascular system (42.7%), drugs targeting blood cells activity and formation (24.6%), and systemic hormonal preparations (16.6%). In detail, renin-angiotensin system inhibitors were commonly used, with ACE inhibitors and/or angiotensin receptor blockers (ARBs) administered either alone or in combination with other cardiovascular therapies. Overall, ACE inhibitors were used in approximately 16% of patients, while ARBs were prescribed in about 15% of patients. Other frequently reported concomitant treatments included beta‑blockers, lipid‑lowering agents and antiplatelet therapies.

**Table 1:** Baseline population features of the GROUND study

Enrolled Population	Statistic	Enrolled (*N* = 199)	Classical (*N* = 169)	Non-classical (*N* = 30)
**Gender**				
Male	n (%)	73 (36.7%)	64 (37.9%)	9 (30.0%)
Female	n (%)	126 (63.3%)	105 (62.1%)	21 (70.0%)
**Age (years)**				
	Mean (SD)	46.7 (16.3)	45.8 (16.2)	51.3 (15.9)
	Median	49.0	48.0	51.0
	Min / Max	18.0 / 82.0	18.0 / 82.0	18.0 / 75.0
**Time from first Fabry disease symptom to diagnosis (months)**				
	25th (95% CI)	8.9 [4.3;24.1]	8.5 [4.2;24.1]	32.7 [1.8; 215.5]
	Median (95% CI)	60.3 [38.8;89.7]	58.0 [38.0;88.6]	190.8 [1.8; 428.4]
	75th (95% CI)	161.1 [113.6;215.5]	153.4 [112.8;206.0]	328.3 [47.3; N.E.]
**Did any relatives suffer of Fabry disease?**				
Yes	n (%)	180 (90.5%)	152 (89.9%)	28 (93.3%)
No	n (%)	10 (5.0%)	8 (4.7%)	2 (6.7%)
Unknown	n (%)	9 (4.5%)	9 (5.3%)	0 (0.0%)
Total n. of relatives suffered/suffer from FD	n (%)	176	148	28
	Mean (SD)	2.5 (1.5)	2.6 (1.5)	2.4 (1.2)
	Median	2.0	2.0	2.0
	Min / Max	1.0 / 9.0	1.0 / 9.0	1.0 / 5.0
**Patients with both available dates**	**n (%)**	**104 (52.3%)**	**96 (56.8%)**	**8 (26.7%)**
Alpha-Galactosidase mutation type*				
Missense/nonsense	n (%)	143 (81.7%)	126 (81.8%)	17 (81.0%)
Splicing	n (%)	2 (1.1%)	1 (0.6%)	1 (4.8%)
Small deletions	n (%)	11 (6.3%)	11 (7.1%)	0 (0.0%)
Unknown	n (%)	10 (5.7%)	8 (5.2%)	2 (9.5%)
Other	n (%)	9 (5.1%)	8 (5.2%)	1 (4.8%)
**α-Galactosidase A activity**				
Absent	n (%)	32 (16.1%)	31 (18.3%)	1 (3.3%)
Reduced	n (%)	76 (38.2%)	64 (37.9%)	12 (40.0%)
Normal	n (%)	35 (17.6%)	24 (14.2%)	11 (36.7%)
Unknown	n (%)	56 (28.1%)	50 (29.6%)	6 (20.0%)
**LysoGb3 level available?**				
Yes	n (%)	86 (43.2%)	73 (43.2%)	13 (43.3%)
No	n (%)	113 (56.8%)	96 (56.8%)	17 (56.7%)
**LysoGb3 value (nmol/L)**	n			
	Mean (SD)	7.9 (9.1)	8.4 (9.6)	5.1 (5.9)
	Median	6.0	6.5	2.2
	Min / Max	0.9 / 70.5	0.9 / 70.5	1.1 / 20.0
**LysoGb3 value (ng/mL)**	n			
	Mean (SD)	6.2 (7.2)	6.6 (7.5)	4.0 (4.6)
	Median	4.7	5.1	1.7
	Min / Max	0.7 / 55.4	0.7 / 55.4	0.9 / 15.7

Globotriaosylsphingosine, LysoGb3; Number, n

### Diagnosis

- As to *genetic and enzymatic patterns*, the most commonly reported *GLA* variants were missense/nonsense (81.7%) in both Cl pts and NCl pts. 143 missense mutations were identified, and 14 Variants of Unknown Significance (VUS) were also reported, of which 6 were defined as benign. Overall, among Cl and NCl cohorts α-Gal A activity was absent in 32 (16.1%) subjects, reduced in 76 (38.2%), normal in 35 (17.6%), and unknown in 56 (28.1%). Moreover, among Cl patients α-Gal A was absent in 18.3%, reduced in 37.9%, normal in 14.2%, unknown/not available in 29.6%, while among NCl patients it was absent in 3.3%, reduced in 40.0%, normal in 36.7%, unknown in 20.0% (*p* = 0.010). LysoGb3 activity was available in 86 (43.2%) individuals and the mean level was 7.9 (SD 9.1) nmol/l and 6.2 (SD 7.2) ng/mL.
- Overall, the most common *onset presentations* were neurological symptoms (44.2%), chronic pain (18.1%), and dermatological signs (32.2%) such as angiokeratomas (13.1%), while the most frequent sign at diagnosis was LVH (73.8%).
- Mean *time from symptom onset to diagnosis* in the 104 (52.3%) pts (*n* = 96 Cl pts, *n* = 8 NCl pts) with both available dates was 5.03 years (95% CI, 38.8 to 89.7 months), more specifically 4.8 years (95% CI, 38.0 to 88.6 months) in Cl pts and 16 years (95% CI, 1.8 to 428.4 months) in NCl pts (*p* = 0.112). Moreover, a median time of 0.64 years (range 0-19.29) from diagnosis to start of FD specific treatment was reported overall.
- *Post-hoc* analysis showed an overall mean *age at FD diagnosis* of 37.9 years (SD 16.20) and a median age of 40.0 years. Among the 153 Tr pts mean *time from diagnosis to beginning of treatment* was 2.29 years (SD 3.80) and median was 0.64 years.
- Overall, the most commonly *involved HCPs* in diagnosis were the cardiologist (128 pts, 64.3%) and the nephrologist (122 pts, 61.3%), with a mean total number of HCPs involved of 2.2 (SD 0.9), while blood DNA test (182 pts, 91.5%) and blood α-Gal A assay (115 pts, 57.8%) were the most used diagnostic tests.

### Treatment trajectories

**Fig. 2:**
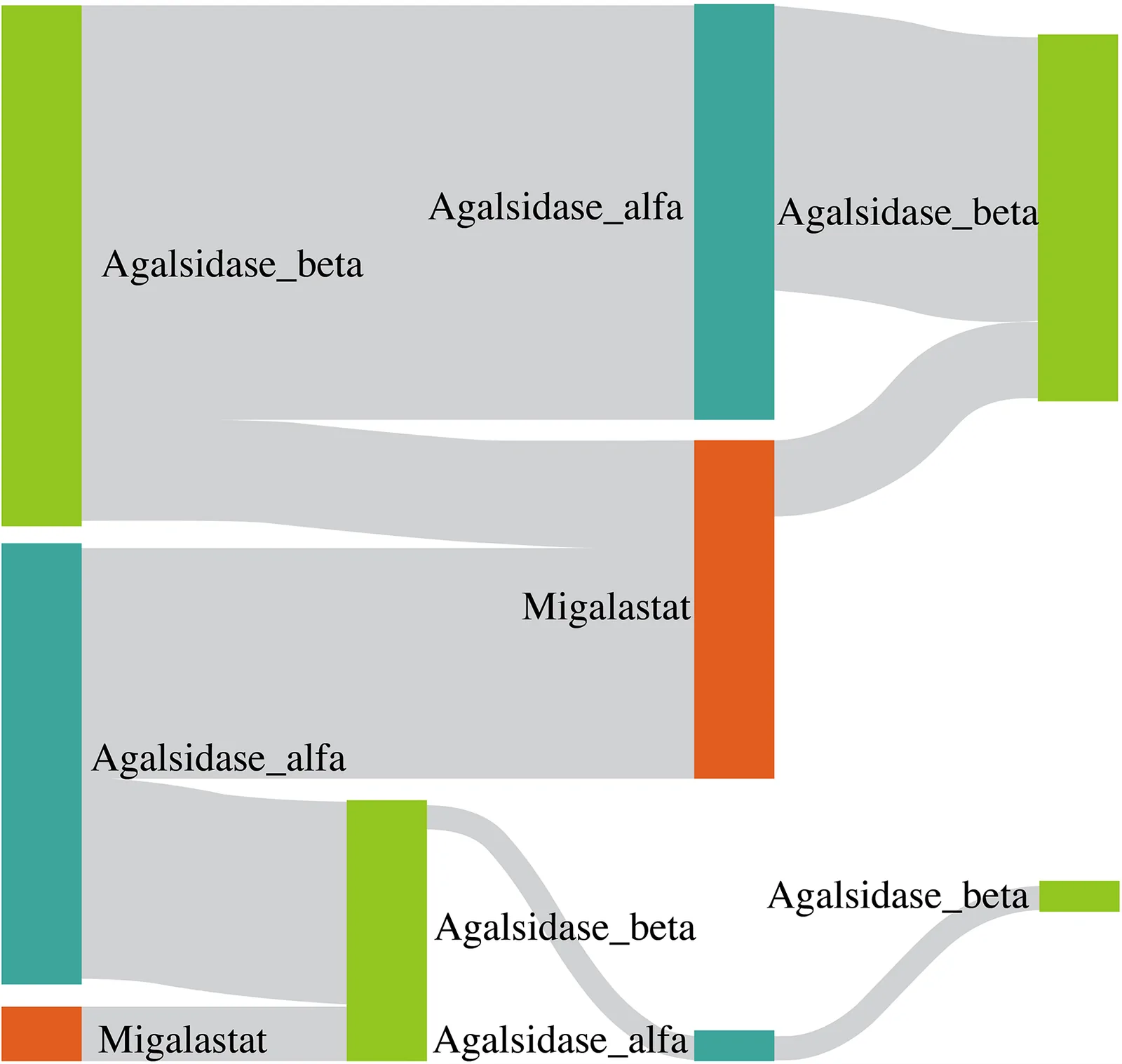
Treatment trajectories as reported in the GROUND study

Overall, 81.9% pts underwent ≥ 1 treatment for FD, i.e., agalsidase alfa (42.4%), agalsidase beta (41.9%), migalastat (15.0%), while 18.1% had never taken any treatment for FD. 30.7% of Tr pts stopped/switched ≥ 1 treatment for FD, due to lack of efficacy (16.9%), side effects (13.6%) and other reasons (69.5%). Permanent treatment discontinuation was observed in approximately 5% of patients mainly related to advanced disease stage or death, persistent adverse events, or patient decision often due to limited perceived benefit or relevant comorbidities. As to phenotype, 87.0% of Cl pts and 53.3% of NCl pts took ≥ 1 treatment for FD (*p* < 0.001). However, differences between the two phenotypes as to switcher and non-switcher proportions, prescribed treatments and reasons for stop/switch were not statistically significant (Fig. 2).

### Primary outcomes

**Fig. 3:**
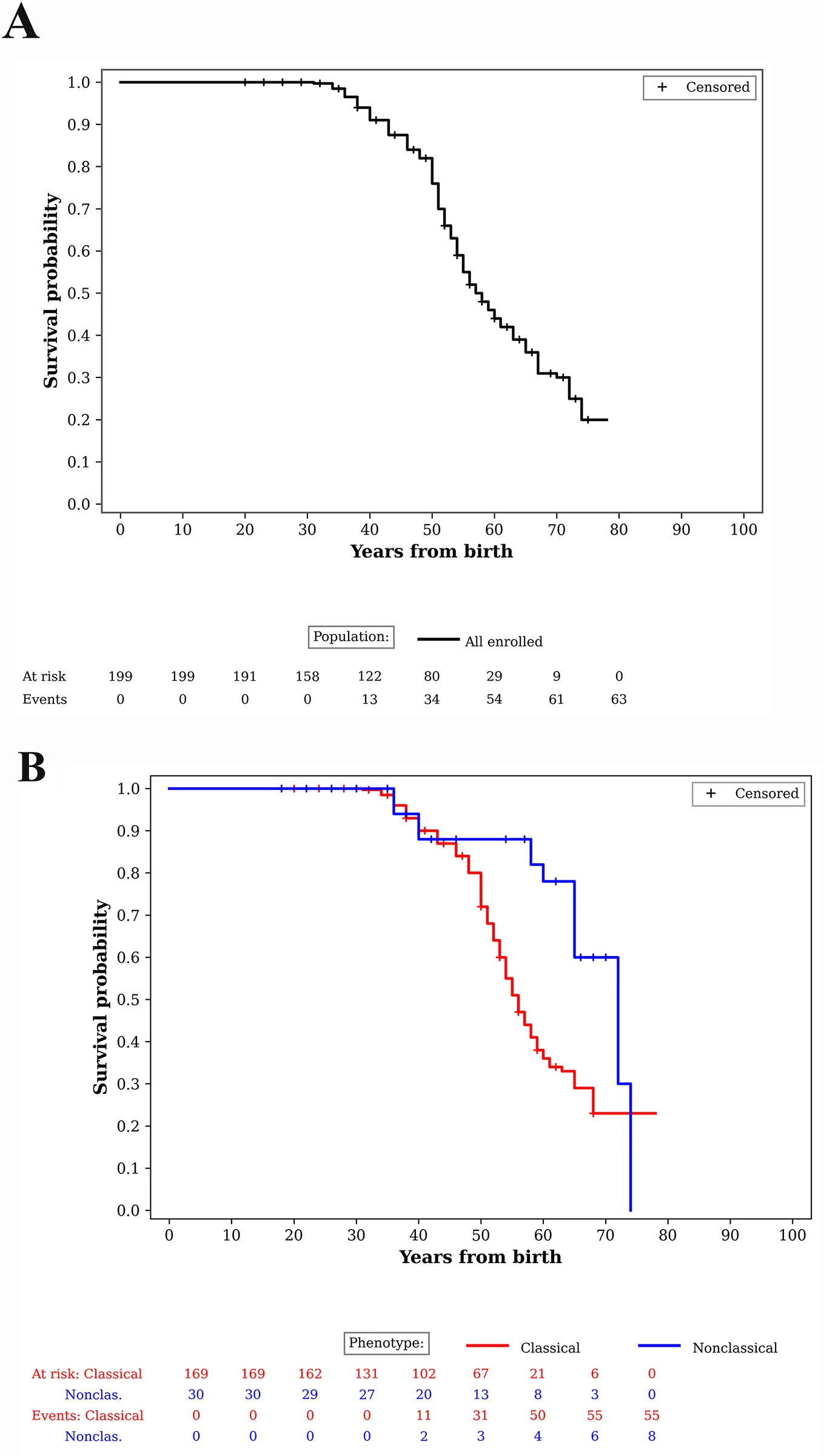
Time to first severe or fatal event, survival function in the enrolled population (**A**) and by phenotype (**B**)

**Fig. 4:**
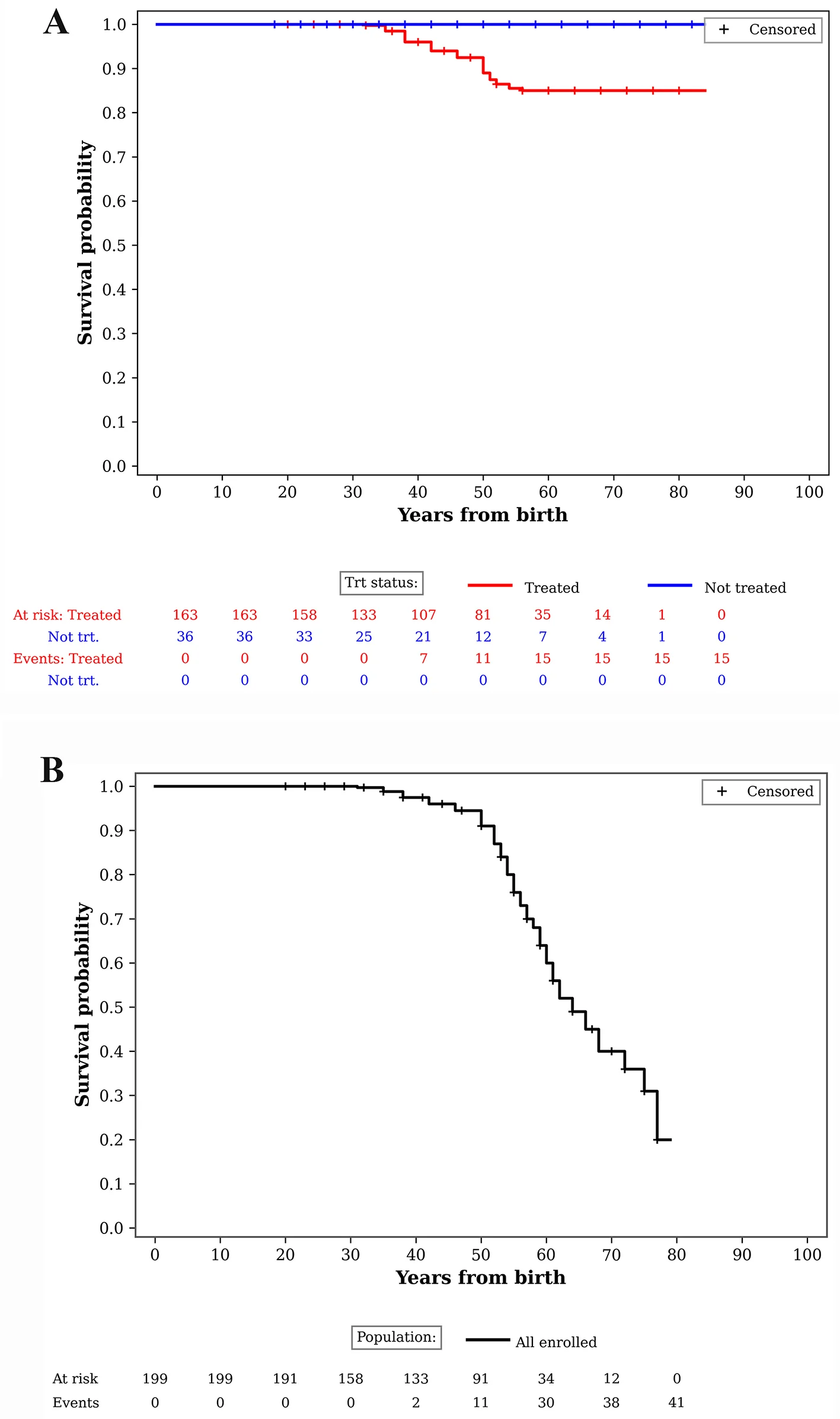

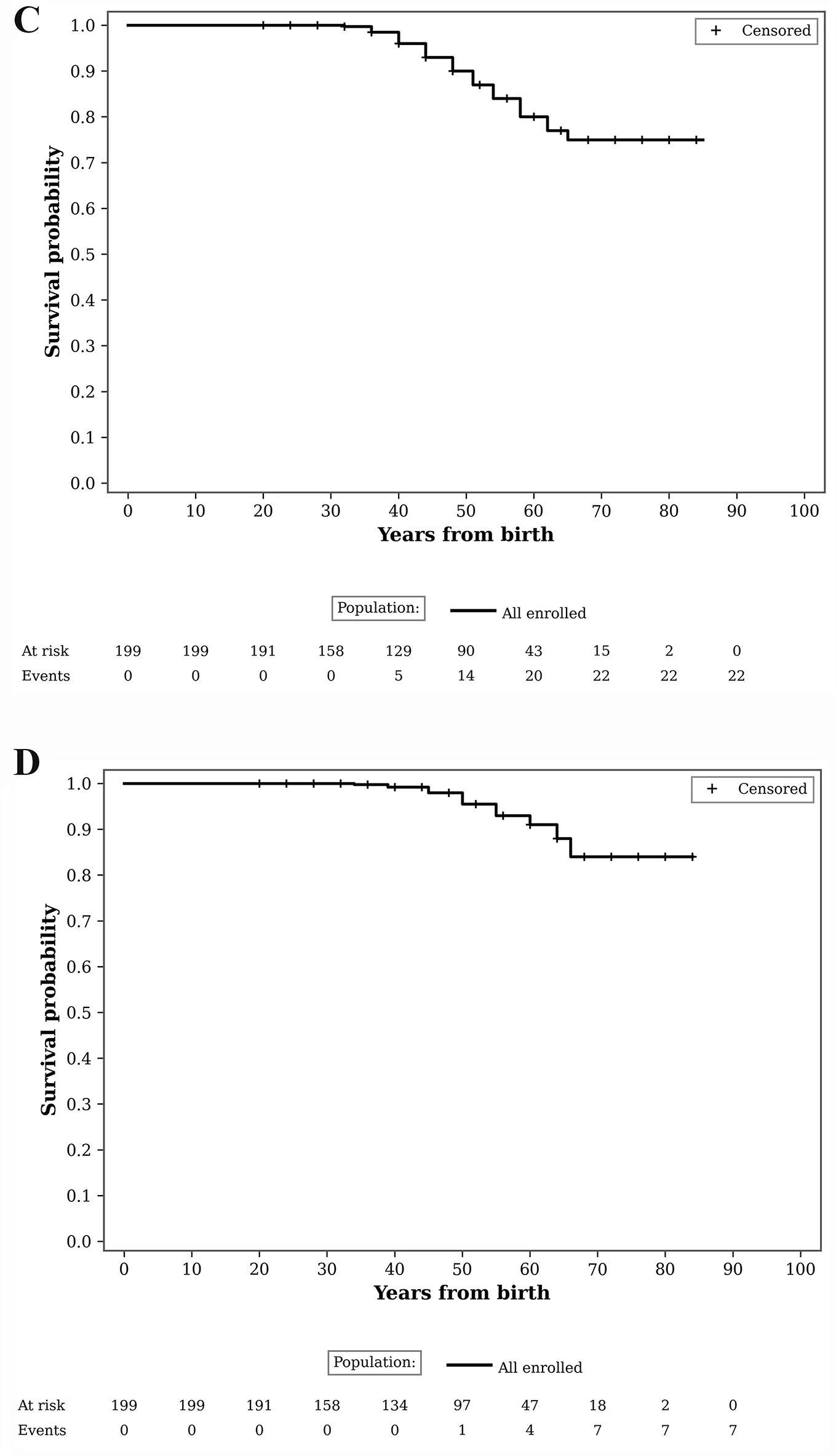
Survival functions (Kaplan-Meyer estimate) as to time to first renal event as to treatment status (**A**); time to first cardiac event in the enrolled population (**B**); time to first cerebral event in the enrolled population (**C**); time to fatal event in the enrolled population (**D**)

- As to the *composite endpoint*, severe or fatal events were reported in 63 (31.7%) pts overall, total number of events was 85 divided as follows: 48% cardiac, 26% cerebral, 18% renal 8% fatal. The median time from birth to the occurrence of a severe or fatal event was 60.0 years (95% CI, 55.0 to 65.0 years). Severe or fatal events were reported in 55 (32.5%) Cl pts and in 8 (26.7%) NCl pts.
- The median time from birth to the occurrence of a severe or fatal event was 57.0 years (95% CI, 54.0 to 61.0 years) in Cl pts and 72.0 years (95% CI, 65.0 years not to evaluable) in NCl pts (Fig. 3).
- As to organ endpoints:
  - Overall, *renal events* were reported in 15 (7.5%) pts all of which were treated (13 males vs. 2 females, 14 Cl pts vs. 1 NCl pt). The median time from birth to the occurrence of a renal event was not evaluable due to the low number of subjects with events, and the difference between phenotypes in the log-rank test was not statistically significant (Fig. 4A).
  - Overall, *cardiac events* were reported in 41 (20.6%) pts (25 males vs. 16 females, 36 Cl pts vs. 5 NCl pts, 38 Tr pts vs. 3 NTr pts). The overall median time from birth to the occurrence of a cardiac event was 65.0 years (95% CI, 60.0 to 74.0 years): 63.0 years (95% CI, 59.0 to 68.0 years) in Cl pts vs. 72.0 years (95% CI, 59.0 years to not evaluable) in NCl pts; 63.0 years (95% CI, 60.0 to 72.0 years) in Tr pts vs. 74.0 years (95% CI, 58.0 years to not evaluable) in NTr pts. However, no statistically significant difference was detected in the log-rank test as to phenotype and treatment *status (*Fig. 4B).
  - Overall, *cerebral events* were reported in 22 (11.1%) pts (10 males vs. 12 females, 19 Cl pts vs. 3 NCl pts, 21 Tr pts vs. 1 Tr pt). The median time from birth to the occurrence of a cerebral event was not evaluable due to the low number of subjects with events (Fig. 4C).
- *Fatal events* were reported in 7 (3.5%) Cl pts (6 males vs. 1 female, 6 Tr pts vs. 1 NTr pts), and they were due to congestive heart failure (*n* = 2), post-surgery complications following kidney transplant (*n* = 2), cardiac event (*n* = 1), chronic kidney failure and sudden cardiac death (*n* = 1), unknown reasons (*n* = 1). The median time from birth to the occurrence of a fatal event was not evaluable due to the low number of pts with events (Fig. 4D).
- *MSSI* was computed in 176 (88.4%) pts (90.5% of Cl pts, 76.7% of NCl pts; 90.2% of Tr pts, 80.6% of NTr pts), with a mean general score of 1.8 (SD 1.8) (median 1.0, range 0 to 8). As to specific organ scores: mean neurological and psychiatric/psychosocial score was 2.3 (SD 2.9) (median 1.0, range 0 to 15); mean cardiovascular score was 4.3 (SD 5.6) (median 1.0, range 0 to 20); mean renal score was 2.6 (SD 4.4) (median 1.0, range 0 to 18); mean total score was 10.9 (SD 10.2) (median 7.0, range 0 to 47). The comparison between phenotypes did not show statistically significant differences for total score and each domain; however, the comparison as to treatment *status* showed statistically significant differences for total and all domain scores (*p* < 0.001), except for neurological and psychiatric/psychosocial score (*p* = 0.036). Mean general score was 1.9 (SD 1.9) in Tr pts vs. 1.2 (SD 0.8) in NTr pts; mean cardiovascular score was 4.9 (SD 5.9) in Tr pts vs. 1.2 (SD 2.7) in NTr pts; mean renal score was 3.0 (SD 4.6) in Tr pts vs. 0.3 (SD 1.0) in NTr pts; mean total score was 12.3 (SD 10.5) in Tr pts vs. 4.2 (SD 4.2) in NTr pts. Outcomes are summed up in Table 2.

**Table 2:** Results of MSSI as the last follow-up/cross-sectional visit (enrolled population)

	Statistic	Enrolled = 199
MSSI computed?		
Yes	n (%)	176 (88.4%)
No	n (%)	23 (11.6%)
General score	n	176
	Mean (SD)	1.8 (1.8)
	Median	1.0
	Min / Max	0.0 / 8.0
Neurological and psychiatric/psychosocial score		
	Mean (SD)	2.3 (2.9)
	Median	1.0
	Min / Max	0.0 / 15.0
Cardiovascular score	n	176
	Mean (SD)	4.3 (5.6)
	Median	1.0
	Min / Max	0.0 / 20.0
Renal score	n	176
	Mean (SD)	2.6 (4.4)
	Median	0.0
	Min / Max	0.0 / 18.0
Total score	n	176
	Mean (SD)	10.9 (10.2)
	Median	7.0
	Min / Max	0.0 / 47.0

### Secondary outcomes

Main outcomes for FASTEX score from diagnosis to the last available measure (i.e., long-term *status*, LT) and from first measure available in the last 1–3 years before LFU/protocol visit to the last available measure (i.e., short-term *status*, ST), were as follows:

- As to *gender*:
  - LT-FASTEX score showed 121 stable pts (overall 66.1%, 70.7% females vs. 58.2% males), and 62 unstable subjects (overall 33.9%, 41.8% males vs. 29.3 females; *p* = 0.086), with a mean FASTEX score change of 26.4 (SD 47.1) (median 0.0, range 0 to 225).
  - ST-FASTEX score showed 110 stable pts (overall 82.1%, 87.3% females vs. 74.5% males), and 24 unstable subjects (overall 17.9%, 25.5% males vs. 12.7% females; *p* = 0.057), with a mean FASTEX score change of 9.1 (SD 23.6) (median 0.0, range 0 to 150).
- As to *predictors of change* in the multivariate analysis of FASTEX score a statistically significant effect was observed for the age (1-year unit: HR 1.039; 95% CI, 1.016 to 1.062; *p* = 0.0008), FD family history (absence vs. presence of FD family history: HR 3.010; 95% CI, 1.001 to 9.044; *p* = 0.0497), and years from diagnosis to LFU (1-year unit: HR 1.058; 95% CI, 1.003 to 1.116; *p* = 0.0377).
- As to *phenotype*:
  - LT-FASTEX score showed that 108 (68.4%) Cl pts and 13 (52.0%) NCl pts were stable, while 50 (31.6%) Cl pts and 12 (48.0%) NCl pts were unstable, mean FASTEX score change was 23.9 (SD 45.3) in Cl pts and 42.2 (SD 55.8) in NCl pts, with no statistically significant subgroup differences for these items.
  - ST-FASTEX score showed that 94 (81.0%) Cl pts and 16 (88.9%) NCl pts were stable, 22 (19.0%) Cl pts and 2 (11.1%) NCl pts were unstable, mean FASTEX score change was 9.7 (SD 24.7) in Cl pts and 4.7 (14.0) in NCl pts, with no statistically significant subgroup differences for these items.
- As to *treatmentstatus*:
  - LT-FASTEX score showed that 99 (64.7%) of Tr pts and 22 (73.3%) of NTr pts were stable, while 54 (35.3%) Tr pts and 8 (26.7%) NTr pts were unstable, mean FASTEX score change was 28.2 (SD 49.1) in Tr pts and 17.3 (34.6) in NTr pts, with no statistically significant subgroup differences for these items.
  - ST-FASTEX score showed that 91 (80.5%) Tr pts and 19 (90.5%) NTr pts were stable, while 22 (19.5%) Tr pts and 2 (9.5%) NTr pts were unstable, mean FASTEX score change was 10.0 (SD 25.1) in Tr pts and 3.8 (SD 11.9) in NTr pts, with no statistically significant subgroup differences for these items.
- As to *decline in renal function* overtime, eGFR slopes in last 1–3 years of FU were − 2.07 (SE: 1.38) and − 0.76 (SE: 1.75) in males and females, respectively.

## Discussion

In the context of a rare and clinically multifaceted disease such as FD, retrospective real-life studies make it possible to assess the effectiveness of patient management from diagnosis to treatment outcomes [20–25]. Therapeutic success in a condition such as FD which features genetic, molecular, phenotypic and clinical complexity depends first and foremost on identifying those patient’s characteristics that drive disease evolution and affect treatment response [26]. Despite the increasing awareness and the availability of targeted genetic tests, different recommendations as to patients’ diagnostic journey, biomarker use and treatment are reported, while patient-intrinsic factors that might influence clinical and therapeutic outcomes are still poorly understood [27].

This retrospective longitudinal cohort study with a cross-sectional phase was performed to assess the clinical burden in terms of severe and fatal outcomes and extension of clinical impairment in the Italian FD population, to identify unmet needs and real-world issues that currently affect FD patient journey from diagnosis to therapy. Despite a predominance of female subjects in the cohort, no baseline statistically significant differences among subgroups by phenotype, treatment and mutation *status*, FD family history, and onset presentation were detected between sexes. The relatively high proportion of females with a classical phenotype likely reflects combined factors, including classification based on clinical manifestations beyond enzyme activity, the impact of X-chromosome inactivation on disease severity, referral bias from specialized Italian Fabry centres, and widespread family screening enabling earlier detection and clearer phenotypic characterization. Lyso-Gb3 was available in about 43% of patients, measured in plasma using local procedures without centralized analysis, mainly at diagnosis and sporadically during follow-up; increases were considered clinically relevant events, but not used alone for treatment decisions, instead acting as a warning sign prompting further evaluation, while limited data and heterogeneity prevented formal assessment of diagnostic accuracy. The median time from first FD symptoms to diagnosis was approximately 5 years, and it was significantly longer in NCl pts. As expected, agalsidase alfa and beta were the most prescribed drugs; however, about 30% of treated patients discontinued or switched therapy, mainly due to perceived lack of efficacy, adverse events, or other factors such as patient preference and logistical burden, with decisions made by multidisciplinary teams; the apparent convergence toward agalsidase-beta reflects real-world trajectories and clinical judgement, while neutralizing antibodies were not routinely assessed and infusion time represented only one of several factors influencing treatment choice. Of note, severe or fatal events were reported in approximately 30% of pts with higher rates in Cl pts and in Tr pts, and the median time from birth to their occurrence was significantly greater in NTr pts. Furthermore, the male gender, a lower age, the absence of a family history of FD and the presence of comorbidities were indicated as predictors of occurrence of a first severe or fatal event. Cardiac, renal and cerebral events showed higher rates in Tr pts, while all fatal events, except one, occurred in Cl and Tr pts, as subjects undergoing therapy are normally those affected by the most severe form of the disease. Mean MSSI score indicated that the enrolled population featured a mild disease, with a more severe impairment as to the cardiovascular component among Tr pts, with no major changes in overall mean eGFR following the first assessment. The rate of stable pts in LT-FASTEX was higher in NTr pts than in Tr pts, unlike ST-FASTEX which showed an increased rate of stable subjects among Tr pts compared to NTr pts. The absence of a family history, an older age and a longer time from FD diagnosis to LFU visit were recognized as predictors of FASTEX score changes in the overall population. Furthermore, significant direct correlations with FASTEX score continuous changes and FASTEX score dichotomous variable (i.e., stable or unstable) were detected for cardiovascular, renal, and total MSSI score. Altogether, the enrolled population presented clinical signs and symptoms of moderate FD, diagnostic delay was less severe than the 10–15 years reported in literature, and disease progression was controlled fairly adequately by the prescribed therapies with good control of renal symptoms, and rates of therapy switch were similar to those reported in current literature [28]. Current worldwide survival data of FD are radically different from those reported in the era before enzyme replacement therapy, when *Branton et al.*. described a cohort of 105 pts in which only 25 survived until the age of 50 and no subject was alive beyond 60 years of age, while other studies reported a reduced life span of about 25 years in males and 10 years in females compared with the general population, and the main cause of death was uremia [29, 30]. However, the presence of subgroups who develop severe complications and events despite treatment, emphasises the need to investigate genetic and clinical features that could influence prognosis as well as the impact of potential benefits of new therapeutic agents in subjects with sub-optimally disease management. Moreover, the use of ACE inhibitors and ARBs in a subset of patients reflects standard clinical practice for the management of cardiovascular and renal comorbidities in FD and underscores the need for an individualized, multidisciplinary approach. Importantly, the low rate of definitive treatment discontinuation observed in this study cohort is consistent with real‑world clinical practice and it was mostly driven by patient‑related factors, rather than lack of efficacy, highlighting the complexity of treatment decisions in FD.

The GROUND study reflects real-world practice without a standardized protocol across centres. However, most patients underwent core diagnostic assessments at baseline (i.e., α-galactosidase A activity, *GLA* testing when consented, renal and cardiac evaluation), while advanced tests were performed based on clinical indication and follow-up was individualized, a variability acknowledged as a limitation, but also reflecting routine Fabry care. In other words, even though this study opens a window on the FD landscape in Italy some issues linked to its design must be emphasized, i.e., data collection uniformity in the enrolled population, the lack of systematic follow-up protocols to monitor all subjects on a standardized basis, and the variability in criteria and timing for FD therapy initiation in relation to an ongoing changing in real-world practice.

## Conclusions

As a systemic disease, FD requires a multidisciplinary patient care and an ongoing collaboration among a variety of specialists. The scientific world has it clear that early treatment can significantly improve prognosis and that currently available therapies, which have radically changed FD clinical history over the last twenty years, are still not optimal for subsets of pts, leaving room for new therapeutic options.

The population of the GROUND study showed a predominance of female over male subjects, Cl pts over NCl pts with most pts treated with ≥ 1 agent due to therapy changes. Overall, diagnosis of FD was reached approximately 5 years from first symptom onset, with a higher delay in NCl pts, while severe or fatal events were reported in about 30% of pts, with higher rates in Cl pts and Tr pts. A significantly greater median time from birth to the occurrence of a severe or fatal event was detected in NTr pts than in Tr pts, possibly suggesting a more severe phenotype in Tr pts that drove treatment start. To support this view, rates of renal, cardiac and cerebral events were higher in Tr pts than in NTr pts and although mean MSSI score indicated a mild disease, the higher mean values for total score and each domain in Tr pts than in NTr pts could suggest that treatments have incomplete efficacy in reducing the severity and preventing the most common complications of the condition. Moreover, clinically significant events were reported in almost 9 out of 10 pts, especially detection/worsening of LVH along with recurrent or new neurological, dermatological and renal/urinary FD-related findings. On the other hand, results of health-related resource consumption indicated that control of the most life-threatening aspects of the disease and acute situations was rather good. Overall results highlight that despite available treatments, FD is still associated with a considerable clinical burden, which calls for a deeper knowledge of those variables that can influence its course and which real-life studies such as this one can help decode.

## Data Availability

The datasets generated and/or analyzed during the current study are not publicly available due to patient confidentiality restrictions but are available from the corresponding author on reasonable request.

## Abbreviations

ANOVA: Analysis of variance
Cl: Classic
CPHR: Cox Proportional Hazards Regression
CSV: Cross-sectional visit
DBS: Dried blood spots
ER: Emergency room
ERT: Enzyme replacement therapies
eGFR: Estimated glomerular filtration rate
FD: Fabry disease
FASTEX: FAbry STabilization indEX
α-Gal A: α-galactosidase A
GI: Gastrointestinal
Gb: 3Globotriaosylceramide
LysoGb: 3Globotriaosylsphingosine
HCPs: Healthcare professionals
KM: Kaplan-Meier
LFU: Last follow-up
LVH: Left ventricular hypertrophy
LT: Long-term
MSSI: Mainz Severity Score Index
NCl: Non-classic
NTr: Non-treated
pts: Patients
QoL: Quality of life
Tr: Treated
ST: Short-term
VUS: Variants of unknown significance
